# Cytological, histological, and molecular characteristics of pure invasive micropapillary carcinoma of pancreas: A case report

**DOI:** 10.1097/MD.0000000000020668

**Published:** 2020-06-12

**Authors:** Sun-Jae Lee, Han-Ik Bae, Ghilsuk Yoon, Chang Min Cho, Hyung Jun Kwon, Jongmin Park, Seung Hyun Cho, An Na Seo

**Affiliations:** a Department of Pathology, Catholic University of Daegu School of Medicine; b Department of Pathology; c Department of Internal Medicine; d Department of Surgery, School of Medicine, Kyungpook National University, Kyungpook National University Chilgok Hospital; e Department of Radiology, Central Physical Examination Office; f Department of Radiology, School of Medicine, Kyungpook National University, Kyungpook National University Hospital; g Department of Radiology, School of Medicine, Kyungpook National University, Kyungpook National University Chilgok Hospital, Daegu, South Korea.

**Keywords:** fine needle aspiration cytology, invasive micropapillary carcinoma, molecular pathology, next-generation sequencing, pancreas

## Abstract

**Introduction:**

Pure invasive micropapillary carcinoma (IMPC) is a rare histologic subtype of pancreatic cancer which has a high propensity for lymph node metastasis and poor prognosis.

**Patient concerns:**

An 81-year-old woman was admitted to our institution with a 3-month history of back pain. Computed tomography of the abdomen and pelvis confirmed the presence of a low-density mass in the tail of the pancreas.

**Diagnosis:**

Endoscopic ultrasound-guided fine needle aspiration cytology (FNAC) from the pancreatic mass showed small tumor cell clusters with three-dimensional aggregates and morula-like structures. The tumor was diagnosed as adenocarcinoma with micropapillary features.

**Interventions:**

The patient underwent radical antegrade modular pancreatosplenectomy and regional lymph node dissection. Histological examination showed small clusters of tumor cells that were closely adhered to one another. The cells were located in empty stromal spaces mimicking lymphovascular channels. All tumor cells showed reverse polarity, resulting in an “inside-out” pattern. An extensive search was performed, and no typical ductal adenocarcinoma component was found. The tumor measured 1.5 × 1.3 cm and invaded into the peripancreatic fat tissue without adjacent organ invasion. One of the 12 regional lymph nodes showed metastasis. Ion Torrent next-generation sequencing identified missense mutations in *KRAS*, *TP53*, and *SMAD4* using the Oncomine Comprehensive Panel version 1.

**Outcomes:**

Twelve months following surgical resection the patient remained healthy with no evidence of recurrence at clinical follow-up.

**Lessons:**

This report highlights the diagnostic features and molecular characteristics of pure pancreatic IMPC and the challenges with diagnosis by FNAC. A centralized and collaborative accumulation of additional cases of pure IMPC could further elucidate its pathogenesis.

## 1 Introduction

Since invasive micropapillary carcinoma (IMPC) was the first described in breast cancer tissue,^[1]^ it has been identified in cancers of several other organs such as the bladder, lungs, salivary glands, and gastrointestinal tract.^[2–7]^ IMPC features has been demonstrated to behave aggressively with a high propensity for lymph node metastases and poor outcomes.^[1–8]^ The pure IMPC subtype is extremely rare, and to the best of our knowledge there is only one previously published case of IMPC, which was located in the head of the pancreas.^[9]^ Herein, we present a likely case of pure IMPC in the pancreatic tail in an 81-year-old woman.

## 2 Case report

This study was approved by the Institutional Review Board of Kyungpook National University Chilgok Hospital (KNUCH), and the informed consent requirement was waived due to the de-identification of all the patient's personal information (No. KNUCH 2019–11–011). All procedures performed in studies involving human participants were in accordance with the ethical standards of the institutional and national research committee and with the 1964 Helsinki declaration and its later amendments or comparable ethical standards.

**Figure 1:**
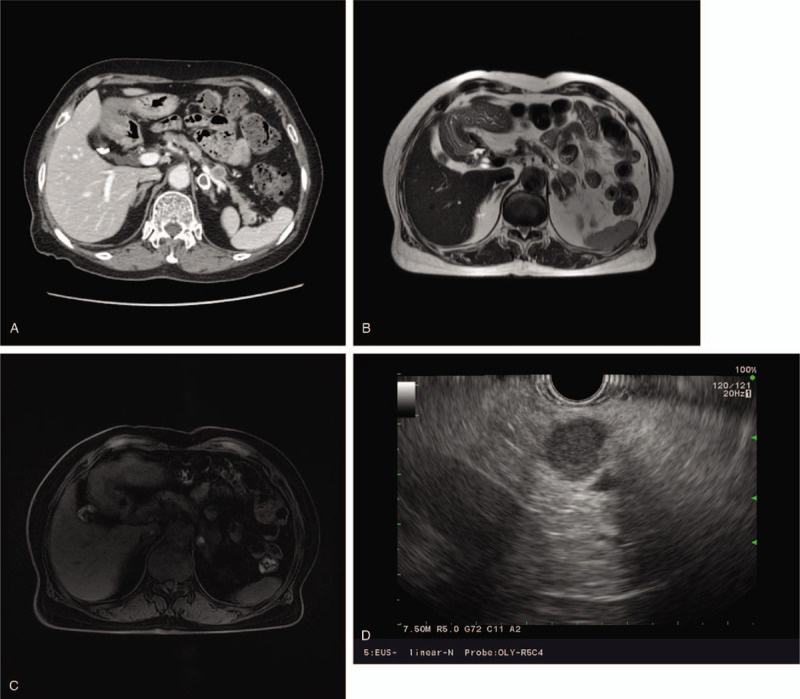
(A) Dynamic computed tomography image of the pancreas shows a mass in the tail of the pancreas mass. Dynamic contrast-enhanced magnetic resonance imaging of the pancreas shows a mass with (B) subtle high signal intensity on T2WI whereas (C) iso signal intensity on T1WI. (D) Diagnostic endoscopic ultrasonography fine needle aspiration of mass was performed.

**Figure 2:**
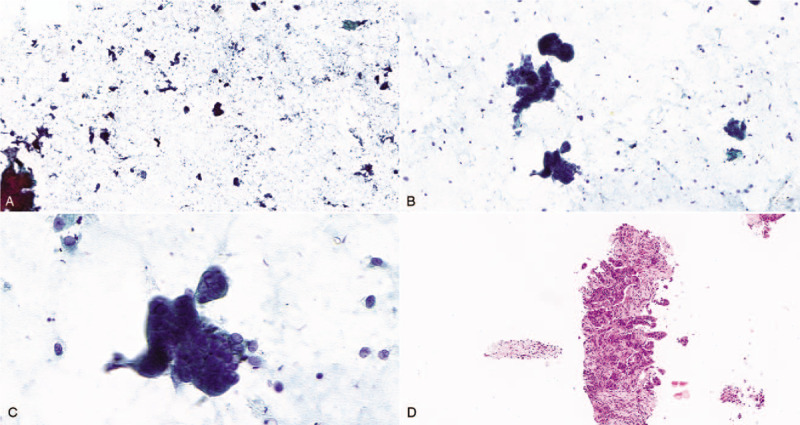
The representative fine needle aspiration cytological findings. Panpanicolaou stained smear showed (A) scattered cells and small tumor groups (×4 objective). (B) The small tumor clusters showed three-dimensional atypical epithelial aggregation and columnar configuration (×20 objective). (C) Tumor cells had high nuclear to cytoplasmic ratio and prominent nucleoli (×60 objective). (D) Hematoxylin- and Eosin-stained cell block showed small tumor clusters with abundant eosinophilic cytoplasm in clear stromal spaces (×10 objective).

An 81-year-old woman suffering back pain over the past 3 months was admitted to our hospital in October 2018. The patient had history of hypertension and diabetes mellitus for several years, which had been treated with standard medications. The patient denied any previous intake of alcohol, tobacco, or herb agent. Preliminary laboratory tests were within normal limits: serum amylase of 51 U/L and serum lipase of 24 U/L. A tumor marker level test revealed mildly elevated levels of Carbohydrate antigen 19-9 (CA 19-9) (39.45 U/mL; normal levels <37). The initial dynamic computed tomography (CT) of the abdomen and pelvis confirmed the presence of a low-density mass in the tail of the pancreas (Fig. 1A). Dynamic contrast-enhanced pancreas magnetic resonance imaging (MRI) was performed with a 3-T system (Discovery 750; GE Healthcare, Milwaukee, WI). The MRI indicated a poorly enhancing mass and no pancreatic duct dilatation. The mass had subtle high signal intensity on T2WI but no signal intensity on T1WI (Fig. 1B and C). Based on the radiologic features, the first impression was suggestive of pseudocyst or ductal adenocarcinoma. To conform the diagnosis, endoscopic ultrasonography fine needle aspiration cytology (FNAC) of the mass was attempted using a 22-gauge needle (EchoTip; Cook Medical Inc, Winston-Salem, NC) (Fig. 1D). The FNAC smears were stained with Papanicolaou stain, and the FNAC cell block was stained with Hematoxylin and Eosin stain. The FNAC smears revealed scattered single columnar cells in the background and small tumor cells clusters with three-dimensional aggregates and morula-like structures. Based on these findings, the mass was diagnosed as adenocarcinoma with micropapillary features (Fig. 2).

**Figure 3:**
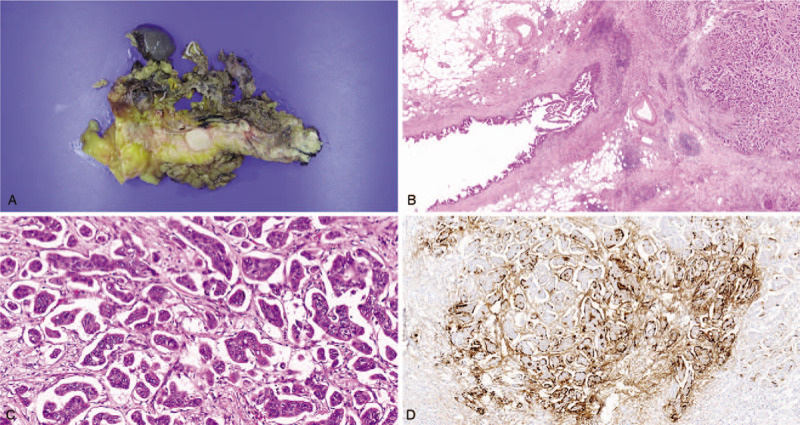
(A) Macroscopically, the tumor was a solid oval lesion of 1.5 × 1.3 cm in size. The representative microscopic features of the tumor are shown. (B) The tumor was entirely composed of numerous small clusters (right side) and was accompanied by an intraductal papillary mucinous neoplasm with intermediate grade (×2 objective). (C) The small clusters of tumor cells surrounded by empty spaces and tumor budding (arrow) were identified in the invasive front of the mass (×20 objective). (D) Immunohistochemical staining for MUC-1 showed a characteristic “inside-out” pattern (×10 objective).

A month later, the patient underwent radical antegrade modular pancreatosplenectomy and regional lymph node dissection. Gross examination showed a solid oval mass measuring 1.5 × 1.3 cm (Fig. 3A). The tumor was yellow in color and had well-demarcated boundaries. No direct tumor invasion was detected in other organs such as the spleen or left adrenal gland. Histological examination of the surgically resected specimen showed small clusters of tumor cells closely adhered to one another. The cell clusters were located in empty stromal spaces mimicking lymphovascular channels (Fig. 3B). All tumor cells showed reverse polarity resulting in an “inside-out” pattern (Fig. 3C). Perineural invasion and lymphovascular invasion were identified, but no evidence of large vessel invasion was observed. An extensive search found no typical ductal adenocarcinoma component. Although intraductal papillary mucinous neoplasm (IPMN) with low grade was concomitant with the IMPC, no correlation or communication with the IMPC was detected. The tumor invaded the peripancreatic soft tissue, and one of the 12 regional lymph nodes showed metastasis. Immunohistochemically, almost all of the tumor cells stained strongly and diffusely for CA 19-9, cytokeratin (CK) 7, and p53, but the stains for DPC-4 (also termed SMAD4) and CK20 were negative. As expected, immunohistochemical staining for MUC1 showed that the stroma-facing surface of the tumor cell cluster was positive (Fig. 3D), confirming the final diagnosis of pure pancreatic IMPC. The patient was discharged from the hospital on day 14. We examined molecular genetic features of this rare tumor via Ion Torrent next-generation sequencing (NGS) technology (ThermoFisher Scientific, MA) using the Oncomine Comprehensive Panel version 1 (ThermoFisher Scientific). We identified missense mutations in *KRAS* (NM_033360.3; C.34G>C), *TP53* (NM_000546.5; c.536A>G), and SMAD4 (NM_005359.5; c.254A>G).

Adjuvant systemic chemotherapy with gemcitabine was started on the 60th post-operative day, given as a biweekly intravenous infusion of gemcitabine for 30 min (900 mg/m^2^). The patient was monitored for recurrence by CT every 3 months. Twelve months following surgical resection the patient remained healthy with no evidence of recurrence at clinical follow-up.

## 3 Discussion

IMPC is a unique histological feature, defined as a carcinoma composed of small clusters of tumor cells within clear stromal spaces that mimic lymphovascular channels.^[1–8]^ IMPC is devoid of fibrovascular cores and the tumor cells exhibit reverse cell polarity (“inside-out” pattern). Common cytologic features of IMPC include:

1. three-dimensional cell clusters with high grade nuclear features, cell balls, morula, staghorn structures, and cell clusters with scalloped borders; and
2. high nuclear/cytoplasmic ratio, dense cytoplasm, moderate to severe nuclear atypia, scattered single cells with columnar configuration, and eccentric nuclei in a mucinous background.^[10]^

As mentioned earlier, the presence of IMPC is associated with more aggressive tumor behavior such as higher frequency of lymph node metastasis and adverse outcomes.^[1–8,10,11]^ Recently, IMPC was officially introduced as a subtype of pancreatic ductal adenocarcinoma in the 5th edition of the WHO tumor classification.^[12]^ Khayyata et al reported 313 patients with pancreatic carcinomas, and eight (2.6%) patients had pancreatic adenocarcinomas in which ≥20% of the mass had an IMPC component.^[13]^ Of the 8 patients, two had tumors with a diffuse micropapillary (MP) pattern (≥80%), four had tumors with a predominant MP pattern (51–80%), and two patients had tumors with a focal MP pattern (20–50%).^[13]^ To the best of our knowledge, there is only one previously published report of pure pancreatic IMPC and two cases of ductal adenocarcinoma with an IMPC component in the English literature to date.^[9,13]^ The clinicopathologic features and behaviors of those tumors as well as the tumor from our case are summarized in Table 1.

**Table 1:**
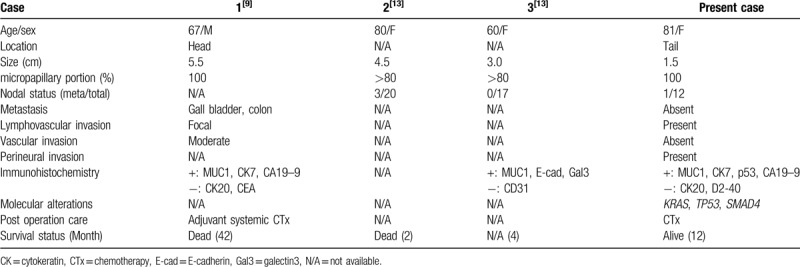
Clinicopathologic characteristics of pure invasive micropapillary carcinoma of pancreas.

Because of its well-known highly metastatic behavior, a pure pancreatic IMPC neoplasm without areas of typical pancreatic cancer must be distinguished from metastatic IMPC from cancers of another organ, such as breast, bladder, other gastrointestinal organs, etc.^[9]^ Unfortunately, the morphology of IMPC is not specific to its location of origin, so the absence of other conventional histologic types in the primary site or in the metastasis does not help with localization of the primary lesion.^[11]^ Immunohistochemical stains can be helpful in identifying the site of tumor origin. With CK7/CK20 immunohistochemical staining, IMPCs of the breast, lung, and parotid gland are CK7+/CK20−, while IMPCs from the colon are CK7−/CK20+.^[9]^ In our case, all evaluated pancreatic pure IMPC showed a CK7+/CK20− pattern and predominant MUC1 expression in the stroma-facing surface of the cell cluster.

There are a few challenges to identifying the differences in molecular characteristics between IMPC and conventional tumor types. The breast IMPC showed specific loss of the 6q16-q22 region, relating to the downregulation of *FOXO3* and *SEC63* gene expression.^[14]^ In bladder urothelial carcinomas, IMPC was strongly associated with *ERBB2* amplification and HER2 overexpression.^[15,16]^ In contrast, colon IMPCs showed significant increase in *TP53* mutation and a trend toward increased *KRAS* and *BRAF* V600E mutations compared to conventional adenocarcinoma.^[11]^ In addition, Lee et al demonstrated increased expression of SOX2 and NOTCH3 as stem cell markers in colon IMPC.^[17]^ In lung adenocarcinomas, *HER2* mutations are observed in the MP features, along with alterations in other genes such as *EGFR, KRAS, ALK*, and *RET*.^[18]^ Notably, we reported the first analysis of the molecular fingerprint of pancreatic IMPC using NGS with an oncology panel. We found hotspot mutations in *KRAS* and *TP53*, which are commonly found in conventional pancreatic ductal adenocarcinoma.^[19]^ However, the molecular characteristic data from our case were limited because we did not sequence the whole genome. Further research is needed to collect more information.

In summary, pure IMPC is a rare neoplasm with a characteristic “inside-out” morphology. Because of its highly aggressive behavior, early detection using FNAC and determination if the lesion is primary or metastatic are both very important. Based on our experience, cytopathologic and molecular findings may help pathologists improve cytologic diagnoses as well as help clinicians determine the pathogenesis and develop targeted therapeutic agents.

## Author contributions

**Conceptualization**: An Na Seo

**Data curation:** Sun-Jae Lee, Han-Ik Bae, Ghilsuk Yoon, Chang Min Cho, Hyung Jun Kwon, Jongmin Park, Seung Hyun Cho, An Na Seo.

**Formal analysis:** Sun-Jae Lee, An Na Seo.

**Funding acquisition**: Sun-Jae Lee

**Investigation:** Sun-Jae Lee, An Na Seo.

**Methodology:** Hyung Jun Kwon, Jongmin Park, An Na Seo.

**Project administration**: An Na Seo.

**Writing – original draft**: Sun-Jae Lee, An Na Seo.

**Writing – review & editing:** Sun-Jae Lee, Han-Ik Bae, Ghilsuk Yoon, Chang Min Cho, Hyung Jun Kwon, Jongmin Park, Seung Hyun Cho, An Na Seo.
